# Commentary: Comparison of different assays for the detection of anticyclic citrullinated peptide antibodies in patients with rheumatoid arthritis

**DOI:** 10.3389/fimmu.2022.1050197

**Published:** 2022-10-24

**Authors:** Sandra Saschenbrecker, Zitao Zeng, Cornelia Dähnrich, Wolfgang Schlumberger

**Affiliations:** Institute for Experimental Immunology, affiliated to EUROIMMUN Medizinische Labordiagnostika AG, Lübeck, Germany

**Keywords:** anti-CCP, anticyclic citrullinated peptide antibodies, chemiluminescence immunoassay (CLIA), diagnosis, electrochemiluminescence immunoassay (ECLIA), enzyme-linked immunosorbent assay (ELISA), immunoturbidimetric assay (ITA), rheumatoid arthritis

## Introduction

In their recent article, Ma et al. evaluated a novel fully automated immunoturbidimetric assay (ITA) developed by Zhejiang Qiangsheng Biological Technology Co. Ltd. for the detection of antibodies against cyclic citrullinated peptide (anti-CCP) in patients with rheumatoid arthritis (RA) (1). Using 131 serum samples from patients with RA, 70 samples from patients with other autoimmune diseases and 63 samples from healthy controls, the diagnostic characteristics of the Qiangsheng assay were compared with three other commercially available assays: [i] EUROIMMUN enzyme-linked immunosorbent assay (ELISA), [ii] Roche Elecsys electrochemiluminescence immunoassay (ECLIA), and [iii] YHLO chemiluminescence immunoassay (CLIA).

We appreciate the objective manner in which the results of the study. However, for reasons stated in this commentary, we are concerned about several interpretations of the results.

## Discussion

Firstly, the authors classify the Qiangsheng citrullinated antigen as a ‘third-generation CCP’ without providing information on the antigen preparation used. As their article is, to our knowledge, the first publication to evaluate the Qiangsheng anti-CCP assay, the characteristics of the antigen and methodological details should be provided to justify its classification as third-generation (anti-CCP3) test equivalent to the Inova QUANTA Lite CCP3 IgG ELISA. Unfortunately, Ma et al. do not address the differences between first-, second- and third-generation anti-CCP tests, but only state that the third-generation Inova ELISA has a higher sensitivity for RA (2). Of note, several studies have demonstrated not only equal or higher sensitivity of the Inova anti-CCP3 assay compared to various anti-CCP2 assays, e.g. the comparative tests in the article, but also similar or worse specificity (3–5).

Secondly, the abstract does not reflect all relevant results of the study: [i] The outcomes of the kappa statistics are limited to the strong qualitative agreement between the assays from EUROIMMUN, Elecsys and YHLO (0.816 ≤ ĸ ≤ 0.863), while it remains unmentioned that the Qiangsheng ITA showed only weak to moderate agreement with these assays (0.582 ≤ ĸ ≤ 0.655). [ii] Based on the ROC AUC values (> 0.9 for each test system, range 0.903 to 0.955) the authors draw the generalized conclusion that all four assays provide similar diagnostic performance for RA. However, they omit to refer to sensitivities and specificities at the manufacturers’ recommended cut-offs, thus concealing the finding that the specificity of the Qiangsheng ITA (74.4%) is substantially lower than that of the comparative tests (96.2-97.0%). Consequently, readers who briefly read the abstract of this article in the belief that it reports the main findings will miss out on important information, resulting in overestimation of the Qiangsheng assay’s diagnostic accuracy.

Similarly, it is suggested in the discussion and conclusion of the article that the Qiangsheng ITA has good diagnostic performance for RA, as do the other three commercial anti-CCP assays. This assessment seems to be based on the comparison of ROC AUC values alone. However, AUC is more of a global measure of assay accuracy that is suitable for general comparative assessment as it summarizes the assay’s discriminative ability over the entire range of cut-offs (6). In contrast, sensitivity and specificity at a given (recommended and experimentally validated) cut-off represent individual assay parameters that are relevant to the diagnostic outcome and, most important, are robustly obtained with registered commercial kits. Strikingly, the specificity of the Qiangsheng ITA was lower than that of the other three assays at similar sensitivities, regardless of whether the results were evaluated based on the manufacturer’s cut-off (ITA: 74.4%, others: 96.2-97.0%) or on the ROC-optimized cut-off (ITA: 81.2%, others: 94.0-96.2%). Considering false positive rates of 42.9% in non-RA patients and 6.3% in healthy individuals, it is incorrect and unacceptable to attest good diagnostic performance to the Qiangsheng ITA. In fact, high specificity of anti-CCP testing is a crucial requirement for the use of these antibodies as an aid in the differential diagnosis between RA and other arthritides that are clinically similar to RA. Although the authors make aware of the low specificity, they do not address the associated negative implications for differential diagnostic conclusions. Instead, at the end of the discussion section, they make a misleading statement about the clinical benefit of a high false positive rate, which carries a high risk of misdiagnosis. Using the Qiangsheng assay for RA screening, as suggested by Ma et al., would require a highly specific secondary assay to reliably identify RA patients. This approach is in turn detrimental to time and cost efficiency.

Finally, the authors point out that ELISAs are difficult to automate due to a long assay time and complex liquid handling procedures, which calls into question their suitability for high-throughput laboratories. The article does not address the fact that the EUROIMMUN ELISA is also suitable for fully automated processing (e.g., on the EUROIMMUN EUROLabWorkstation or EUROIMMUN Analyzer I) with the parameters as given in Table 1 by Ma et al., thus accomplishing high-throughput testing with up to >200 results per hour as well. The description of the study methods does not specify whether ELISA testing was carried out manually or automatically, while for the other three assays the automation platforms used are explicitly stated (Qiangsheng ITA: Beckman Coulter AU5800 Clinical Chemistry Analyzer; Elecsys ECLIA: Roche Modular Analytics E170; YHLO CLIA: YHLO iFlash 3000 chemiluminescence immunoassay Analyzer).

## Author contributions

WS had the idea for this commentary. ZZ carried out analyses on the Chinese IVD market. SS wrote the first draft of the manuscript. CD and WS contributed diagnostic and technical expertise to discussions. All authors have made a substantial, direct, and intellectual contribution to the work and approved the submitted article version for publication.

## Conflict of interest

SS, ZZ, CD and WS are employees of EUROIMMUN, a manufacturer of diagnostic reagents.

## Publisher’s note

All claims expressed in this article are solely those of the authors and do not necessarily represent those of their affiliated organizations, or those of the publisher, the editors and the reviewers. Any product that may be evaluated in this article, or claim that may be made by its manufacturer, is not guaranteed or endorsed by the publisher.
